# Utrogestan as an Effective Oral Alternative for Preventing Premature Luteinizing Hormone Surges in Women Undergoing Controlled Ovarian Hyperstimulation for In Vitro Fertilization

**DOI:** 10.1097/MD.0000000000000909

**Published:** 2015-05-29

**Authors:** Xiuxian Zhu, Xiaole Zhang, Yonglun Fu

**Affiliations:** From the Department of Assisted Reproduction, Shanghai Ninth People's Hospital, Shanghai Jiaotong University School of Medicine (XZ, YF); Department of Gynecology, Shuguang Hospital Affiliated to Shanghai University of TCM, Shanghai, China (XZ).

## Abstract

A major cause of cycle cancellation during controlled ovarian hyperstimulation (COH) in women undergoing in vitro fertilization (IVF) is the occurrence of premature luteinizing hormone (LH) surges. Steroidal preparations can modulate the secretion of gonadotropins (Gn); however, few studies using progesterone to inhibit the premature LH surges in COH have been published. The purpose of the study was to evaluate the oral delivery of progesterone soft capsules (Utrogestan) to prevent LH surges from the follicular phase and to compare cycle characteristics as well as to evaluate pregnancy outcomes in subsequent frozen-thawed embryo transfer (FET) cycles.

A total of 374 patients were enrolled in this retrospective study, among which 187 patients were simultaneously administered Utrogestan and human menopausal gonadotrophin (hMG) from cycle day 3 until the trigger day. A short protocol including 187 controls with comparable age, body mass index (BMI), infertility duration, and antral follicle count was also used. GnRH agonist (0.1 mg) or hCG (3000 IU) was used for a trigger when the dominant follicles matured. Viable embryos were cryopreserved for later transfer in both groups. The primary outcome was the number of oocytes retrieved. The secondary outcomes included the number of mature oocytes, incidence of premature LH surge, and clinical pregnancy outcomes from FET cycles.

Consistent LH suppression was achieved during COH, with a range of 0.07 to 8.9 IU/L, and no premature LH surge was detected. The number of oocytes retrieved in the Utrogestan and hMG protocol was comparable with that in the short protocol (10.92 ± 5.74 vs 10.6 ± 6.22, *P* > 0.05), and the dose of hMG was higher than that used in the short protocol (1884.22 ± 439.47 IU vs 1446.26 ± 550.48 IU, *P* < 0.05). No significant between-group difference was observed in the mature oocyte rate (88.88% vs 90.12%), cleavage rate (96.58% vs 96.58%), clinical pregnancy rate (54.27% vs 51.65%), or implantation rate (33.59% vs 34.02%).

The study shows that Utrogestan is an effective oral alternative for preventing premature LH surges in women undergoing COH, which will help to establish a convenient user regimen in combination with FET.

## INTRODUCTION

The occurrence of premature luteinizing hormone (LH) surges has been sharply reduced since the introduction of gonadotropin-releasing hormone agonist (GnRH-a). However, there are still some disadvantages, such as the complexity of achieving consistent downregulation, an increased risk of ovarian hyperstimulation syndrome (OHSS) from a human chorionic gonadotrophin (hCG) trigger, and expensive cost, which has prompted interest in exploring convenient alternatives to prevent premature LH surges.^1,2^

We previously demonstrated that no premature LH surges were detected during luteal-phase ovarian stimulation.^3,4^ Perhaps the high doses of progesterone results in pituitary suppression, which is aligned with Letteries’ pilot finding that a combination of ethinyl estradiol and norethindrone was restricted to a 5-day course beginning on day 6 or 8 and permitted folliculogenesis, but effectively inhibited mid-cycle LH surges and ovulation during in vitro fertilization (IVF) stimulation.^5^ The preliminary data suggest that steroidal preparations may be an inexpensive and effective method of preventing LH secretion.

The usage of steroidal preparations could not be considered because of its negative impact on endometrial receptivity when fresh embryo transfers were routinely performed during IVF in the past several decades. However, frozen-thawed embryo transfer (FET) has been widely used recently, with the safety of cryopreservation techniques being confirmed, as well as the advantages of the “freeze-all” strategy, which can increase cumulative pregnancy rates, decrease multiple pregnancy rates and ectopic pregnancy rates, and reduce the risk of OHSS.^6–8^ The “freeze-all” strategy made steroidal preparations in IVF possible.

We hypothesized that the progesterone delivered from the early follicular phase may be used to suppress premature LH surges in IVF cycles. Progesterone soft capsules (brand name: Utrogestan), as natural micronized progesterone, are usually used for luteal support and was orally delivered from menstruation cycle day 3 (MC3) until the trigger day to mimic the high context of progesterone in the luteal phase, which can be detected in serum after being taken orally or vaginally.^9,10^ To the best of our knowledge, there is no report using Utrogestan to achieve the suppression of LH. We conducted a retrospective study to explore the novel use of Utrogestan combined with human menopausal gonadotropin (hMG) in controlled ovarian hyperstimulation (COH) during IVF, aiming to compare the endocrine characteristics, embryological characteristics, and pregnancy outcomes in FET cycles compared with a short protocol.

## MATERIALS AND METHODS

### Study Setting and Patients

A retrospective study was conducted at the Department of Assisted Reproduction at the Ninth People's Hospital of Shanghai Jiaotong University School of Medicine. Women undergoing IVF/intracytoplasmic sperm injection (ICSI) regimens for infertility treatment were recruited from June 2013 to July 2014. The protocol was approved by the Ethics Committee (Institutional Review Board) of the Ninth People's Hospital of Shanghai. The study was conducted according to the Declaration of Helsinki for medical research. All participants provided informed consent after counseling for infertility treatments and routine IVF procedures.

The following inclusion criteria were used: no older than 38 years of age; regular menstrual cycles over the previous 3-month period (25–35 days); an antral follicle count of more than 4 on MC2–3; and a basal serum follicle stimulating hormone(FSH) concentration no higher than 10 IU/L.

The following exclusion criteria were applied: documented ovarian failure, including a basal FSH level above 10 IU/L or no antral follicles, according to ultrasound examination; endometriosis grade 3 or higher; a diagnosis of polycystic ovarian syndrome (PCOS); administration of hormonal treatments in the previous 3 months; any contraindications to ovarian stimulation treatment; and documented cycles with no oocyte retrieved.

### Procedures

#### Controlled Ovarian Stimulation and Allocation

The study group was administered hMG 150 to 225 IU (Maanshan Pharmaceutical Trading Co., Anhui, China) and Utrogestan (Laboratories Besins International, Paris, France) 100 mg twice a day from MC3 until the trigger day. Follicular monitoring started at MC9–11 and was performed every 2 to 4 days using a transvaginal ultrasound examination to record the number of developing follicles. Serum FSH, LH, E2, and progesterone concentrations were measured using patient blood tests on the same days as the ultrasound exams. When 3 dominant follicles reached 18 mm in diameter, the final stage of oocyte maturation was triggered using triptorelin 0.1 mg (Decapeptyl, Ferring Pharmaceuticals, Germany) or hCG 3000 IU (Lizhu Pharmaceutical Trading Co., Zhuhai, China). Transvaginal ultrasound-guided oocyte retrieval was conducted 34 to 36 hours after the trigger. All follicles with diameters of more than 10 mm were retrieved.

A short protocol was used for the control group. Patients were administered triptorelin 0.1 mg daily beginning on MC2 and hMG 150 to 225 IU daily beginning on MC3. After 6 to 7 days, the ultrasound examination and serum hormone level tests were performed, and the hMG dose was adjusted, according to the follicle development. When the dominant follicles reached 18 mm in diameter, the final stage of oocyte maturation was induced with an intramuscular injection of hCG 3000 IU. Oocyte retrieval was conducted 34 to 36 hours later. Fertilization of the aspirated oocytes was performed by either IVF or intracytoplasmic sperm injection (ICSI), depending on the semen parameters. According to the number and regularity of blastomeres and the degree of embryonic fragmentation, good-quality embryos (including grade 1 and grade 2, 8-cell embryos) were frozen by vitrification on the third day following the oocyte retrieval, and non-top-quality embryos were placed in extended culture, of which good morphological grade blastocysts were frozen on day 5 or day 6.

#### Endometrium Preparation and FET

In this study, the method of endometrium preparation was similar in both groups. Specifically, natural cycle was used for the women with regular menstrual cycles, letrozole was employed for the women with irregular menstrual cycles, and hormone replacement treatment (HRT) was recommended for patients with thin endometrium during either natural cycles or stimulation cycles. HRT referred to the oral administration of ethinyl estradiol 25 μg tid (Xinyi Pharmaceutical Co., Shanghai, China) from MC3 onwards. The transfer of day 3 embryos or blastocyst was scheduled based on the embryo and endometrium synchronization. When pregnancy was achieved, the progesterone supplement was continued until 10 weeks of gestation.

#### Hormonal Measurement

Serum FSH, LH, E2, and progesterone were collected on day 3 of the stimulation cycle, MC 9–11 (after 6 to 8 days of stimulation), the trigger day and the day after the trigger (about 10 hours later after the injection of GnRH-a or hCG). Hormonal levels were measured with chemiluminescence (Abbott Biologicals B.V., Netherlands). The lower sensitivity limits were as follows: FSH=0.06 IU/L, LH = 0.09 IU/L, E2 = 10 pg/mL, and *P* = 0.1 ng/mL. The upper limit for the E2 measurement was 5000 pg/mL. The E2 values were recorded as 5000 pg/mL if the E2 levels on the trigger day or the day after the trigger were higher than the upper limit.

### Statistical Analysis

The primary outcome measure was the number of oocytes retrieved. The secondary measures included the clinical pregnancy rate, ongoing pregnancy rate, and FET implantation rate. Clinical pregnancy was defined as the presence of a gestational sac with fetal heart activity during ultrasound examination 7 weeks after FET. The implantation rate was defined as the number of gestational sacs divided by the number of embryos transferred. The miscarriage rate was defined as the proportion of patients with spontaneous termination of pregnancy. The cycle cancellation referred to the patients who completed the oocyte retrieval without viable embryos.

**FIGURE 1:**
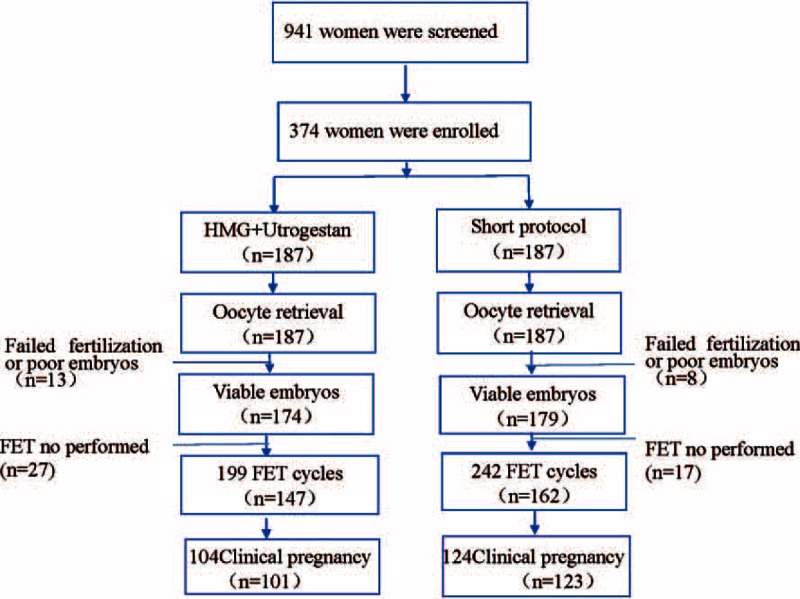
The flowchart of the study.

In the table presented in this study, the data are presented as the mean ± SD, and in Figure 1, the hormone profile is presented as the mean ± SEM. The data were analyzed using one-way analysis of variance, using the Bonferroni method and Dunnetts’ test as appropriate, and *P* < 0.05 was considered to be statistically significant. All data were analyzed using the Statistical Package for the Social Sciences for Windows software (version 16.0, SPSS Inc.).

## RESULTS

### Patient Characteristics

Figure 1 shows a profile summary of the study. A total of 941 women were screened for the study, including 187 women in the study group (Utrogestan + hMG protocol) and 187 women in the control group (a short protocol). The remaining 567 women were rejected in accordance with the predefined study exclusion criteria. A total of 353 women completed oocyte retrieval cycles, and 309 women completed FET cycles. All participants succeeded in producing oocytes (range, 1 to 36), and 353 women (94.39%) had the highest quality embryos to cryopreserve, while 21 patients were excluded from the study because they did not produce the highest quality embryos.

There was no significant between-group difference among the 2 groups in terms of age, BMI, number of antral follicles, duration of infertility, and basal endocrine characteristics (*P* > 0.05). No difference was observed in the proportion of indications, including tubal factor, male factor, combination of tubal and male factor, and unknown factor. In the study, 20.32% (38/187) of the study group patients and 15.51% (29/187) of the control group participants had previously failed FET treatments (Table 1).

**TABLE 1:**
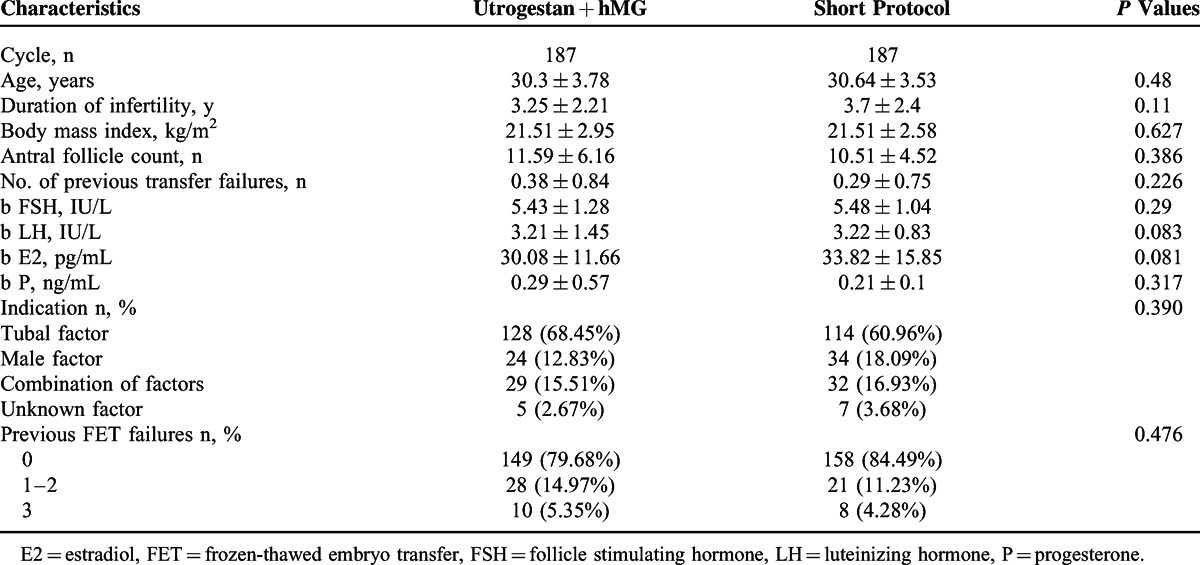
General Information of Patients (x¯±*S*)

### Ovarian Stimulation, Follicle Development, and Oocyte Performance

All women in both groups had 1 to 36 oocytes retrieved. Thirteen women in the study group and 8 women in the control group had either no fertilized oocytes or poor-quality embryos. A total of 353 women across the groups had 1 to 14 embryos cryopreserved after their oocyte retrieval. Table 2 describes the clinical and cycle characteristics of COH in both groups. The mean stimulation duration and doses of hMG, number of follicles with a diameter larger than 10 and 14 mm, fertilization rate, and viable embryo rate per oocyte retrieved were significantly higher in the study group than in the control group (*P* < 0.05). The number of viable embryos in the study group was slightly higher, reaching the level of statistical difference compared with the short protocol (4.99 ± 2.51 vs 4.45 ± 2.46, *P* < 0.05). No significant between-group difference was found in the number of oocytes retrieved, MII oocytes, fertilized oocytes, cleaved embryos, D3 top-quality embryos and the rate of oocyte retrieval, oocyte maturation, and cleavage (*P* > 0.05). The cycle cancellation rate due to no viable embryos did not differ between the 2 groups (6.95% vs 4.28%, *P* > 0.05). No patient experienced moderate or severe OHSS during the study.

**TABLE 2:**
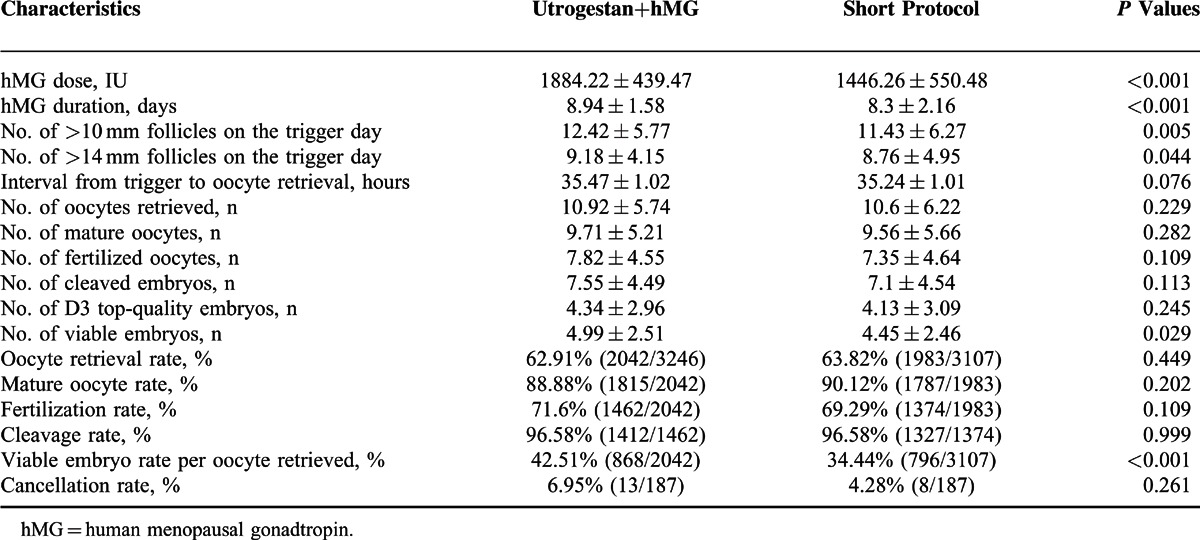
The Stimulation and Embryological Characteristics of the Patients (x¯±*S*/%)

### Pregnancy

In our study, 309 women completed a total of 441 FET cycles, including 204 women who underwent 1 FET, 82 women who completed 2 FETs, 19 women who finished 3 FETs, and 4 women who completed 4 FET cycles. The remaining 44 women did not complete their FET cycles for personal reason before the end of the study. Ninety-three women completed postnatal outcome follow-ups after their FETs (Table 3).

**TABLE 3:**
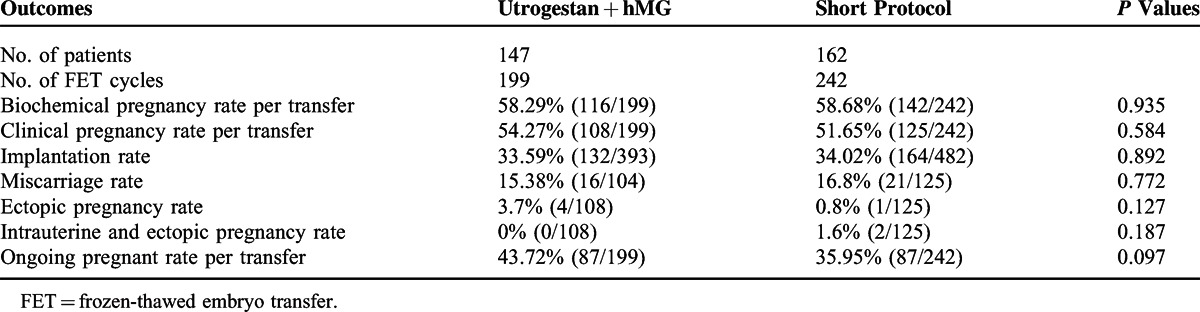
Pregnancy Outcomes of Frozen-Thawed Embryos Originating From the 2 Regimens

A total of 466 embryos were thawed, and the rate of viable frozen-thawed embryos was 98.5% (459/466). The clinical pregnancy rate per transfer was 54.27% (108/199) in the study group, in contrast with 51.65% (125/242) in the short protocol group. Note that 15.38% (16/104) of the patients in the study group had a miscarriage before reaching the gestational age of 12 weeks, while 16.8% (21/125) of patients in the short protocol group miscarried. The implantation rate in the study group was similar to that in the short control group (33.59% vs 34.02%) (*P* > 0.05), which indicated that the embryos in both groups shared similar development potential.

Of all pregnancies in the study group, 13 women had live births and 74 had ongoing pregnancies in the study group compared with 80 live births and 7 ongoing pregnancies in the control group by the end of the research period. Delivery follow-up showed 5 twin births and 8 single births in Utrogestan + hMG protocol in contrast with 16 twin births and 64 single births. No malformations were reported in the newborns. The mean newborn birth weight was 3214.71 ± 515.68 g (range, 2300–4050 g) and the mean birth height was 49.71 ± 1.19 cm (range, 43–52 cm) in the study group while the mean newborn birth weight was 3244.81 ± 497.33 g (range, 2025–4350 g) and the mean birth height was 49.73 ± 1.17 cm (range, 46–53 cm). All newborns were healthy during follow-up. The Utrogestan + hMG protocol was tried steadily, and the new protocol was applied widely since 2014, leading to most of the pregnant patients was ongoing.

### Analysis of Utrogestan + hMG Protocol in the Subgroups of Trigger by GnRH-a or hCG

In this trial, GnRH-a and hCG were used to trigger final oocyte maturation in the study group. Table 4 describes the cycle characteristics in the subgroups of trigger by GnRH-a or by HCG. The viable embryo rate per oocyte retrieved after trigger by GnRH-a was significant different with that after trigger by hCG (40.12% vs 45%, *P* < 0.05). No difference was found in the other cycle characteristics of the 2 subgroups.

**TABLE 4:**
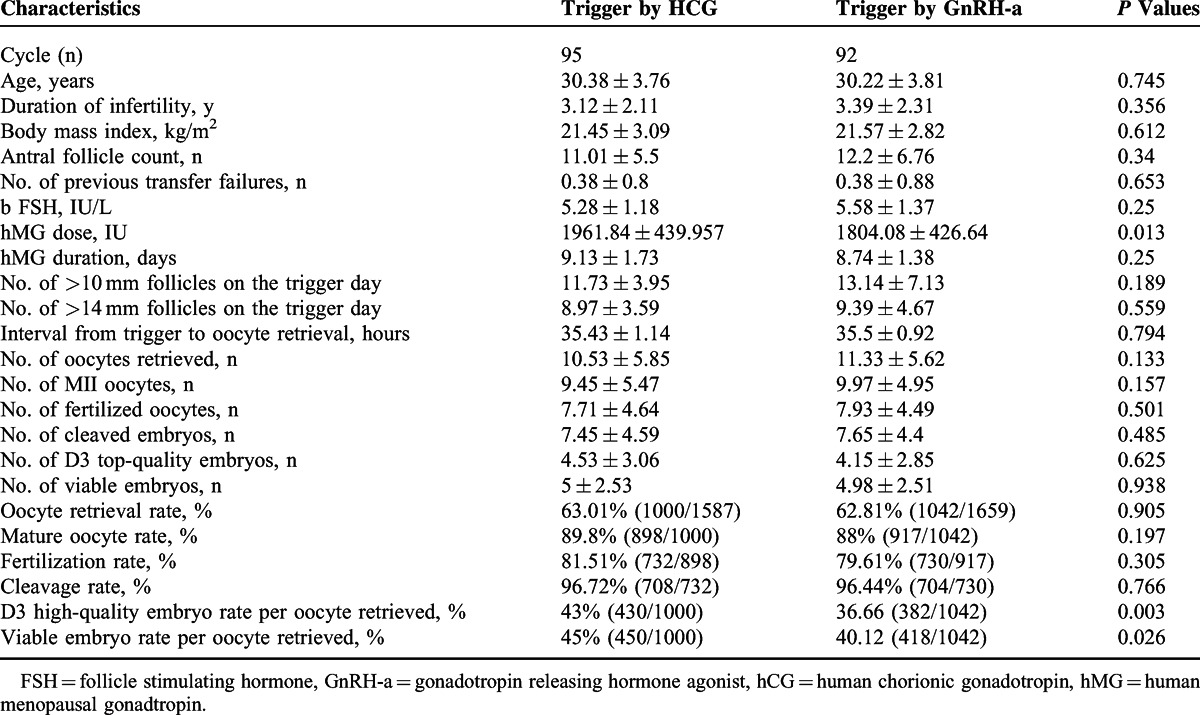
The Cycle Characteristics in the Subgroups of Trigger by GnRHa or by HCG

**FIGURE 2:**
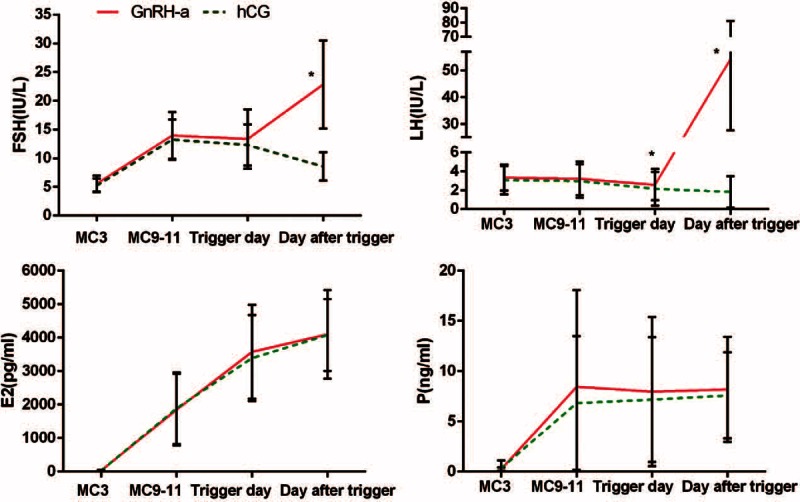
The hormone profiles of Utrogestan + hMG protocol in the subgroups of trigger by GnRH-a or hCG. The mean ± SD values show the temporal associations among circulating concentrations of FSH, LH, E2, and P. The red line refers to the subgroup with GnRH-a trigger and the green line stands for the subgroup with hCG trigger. The asterisk (∗) represent *P* < 0.05 at the time point.

As the different response of the pituitary after trigger by GnRH-a or hCG, the hormone profiles of Utrogestan + hMG protocol were drawn separately according to the way to trigger (Figure 2). FSH levels increased significantly after hMG administration and kept steady during ovarian stimulation. After trigger by GnRH-a, FSH increased to 22.83 ± 7.66 IU/L, in contrast, the FSH level on the day after trigger by hCG was 8.56 ± 2.45 IU/L (*P* < 0.05). The LH values gradually decreased during ovarian stimulation with a range of 0.07 to 8.9 IU/L. The LH level on the trigger day in GnRH-a was significantly lower than that by hCG (2.15 ± 1.78 vs 2.59 ± 1.67, *P* < 0.05), and then increased significantly to 54.36 ± 26.74 IU/L at the time of 10 hours later after trigger by GnRH-a compared with 1.82 ± 1.65 IU/L by hCG (*P* < 0.05). Serum E2 values increased gradually with the growth of follicles during ovarian stimulation, with no significance in the 2 subgroups. Serum *P* values increased after the delivery of Utrogestan, with a scope of 1.1 to 37.57 ng/mL, and maintained at a stable concentration; however, there was no significant difference of progesterone values between the 2 subgroups.

## DISCUSSION

In this study, Utrogestan was used to prevent premature LH surges during COH in combination with the freeze-all strategy. Hormonal measurements showed that the pituitary LH levels were suppressed after a 6-day Utrogestan treatment, and no premature LH surge was observed during COH. To the best of our knowledge, these data firstly demonstrated that Utrogestan effectively inhibited LH.

The rates of oocyte retrieval, mature oocytes, and cleaved embryos retrieved, and the pregnancy results after FET in the study group were similar to those in the short protocol. Moreover, the viable embryo rate per oocyte in the study group was higher than that in the control group. Therefore, it was demonstrated that the Utrogestan and hMG protocol was feasible for producing competent oocytes/embryos. However, the duration of hMG administration and the hMG dose were higher in the study group than in the control group, a finding that is consistent with prior studies of luteal-phase ovarian stimulation.^3,4^ One possible reason for this finding is associated with the extent of pituitary suppression during COH. The LH level on the trigger day in the study group was significantly lower than that in short protocol. In addition, it is possible that the environment of high progesterone has a direct influence on the function of ovarian granulosa cells.

Previous experiments provide the foundation of the novel usage of Utrogestan. As described by Dierschke et al, when circulating estrogens and progesterone (P) increased simultaneously or when P was introduced 12 hours after the estradiol benzoate (EB) injection, acute elevations in plasma P concentrations to supraphysiological levels could block the discharges of LH in rhesus monkeys.^11^ In addition, according to Richter's research in ewes, induction of the GnRH surge by estradiol can be differentiated into the following 3 stages: activation, transmission, and the GnRH surge itself, which stimulates an LH surge.^12–14^ Progesterone can block the estradiol-induced surge-generating signal soon after the onset of signal transmission (immediately after estradiol removal) but not during the later stages of signal transmission (at the time of GnRH/LH surge onset).^15–17^ Therefore, progesterone is capable of facilitating or blocking the LH surge, depending on the timing of its administration.^18^ In our preliminary trial, LH surge blockage failed if Utrogestan administration was started when the diameter of multiple follicles was more than 10 mm, according to the method of adding GnRH antagonists (data unpublished). Therefore, we modified the regimen by using Utrogestan from MC3.

The association between the extent of hypothalamic-pituitary-ovarian (HPO) axis suppression and the dose of progesterone administration was not addressed in the trial design. A dose of 200 mg was attempted in our trial, because previous studies have suggested that mean plasma levels can reach as high as luteal phase levels at doses of more than 100 mg.^9,10^ The elevated progesterone values were not due to the spontaneous ovulation, because no luteinized cysts were detected throughout the COH. One explanation is that exogenous natural progesterone (Utrogestan) may exert an autoregulatory positive feedback action to enhance the production of endogenous progesterone. It has been demonstrated in rat granulosa cells that progestins can stimulate progesterone biosynthesis via enhancing the 3β-hydroxysteroid dehydrogenase (3β-HSD) enzyme.^19–21^ In future, we perhaps can tailor the dose of progesterone in terms of the concentration of P and LH.

The mechanism underlying the process of progesterone blocking the E2-induced GnRH surge in females is unknown. Some studies proposed that the effect of progesterone is mediated by the classic progesterone nuclear receptors (PR).^22^ Recently, certain factors, such as endogenous opioid peptides (EOPs), progesterone receptor membrane component 1 (PgRMC1), and periventricular preoptic area (pePOA) neurons, were proven to play a role in progesterone negative feedback on pulsatile GnRH secretion.^23–25^

Previous studies have reported that high progesterone levels had an adverse effect on the clinical pregnant rate.^26^ Based on the meta-analysis of more than 60,000 cycles, Venetis et al confirmed that the detrimental role of progesterone in fresh IVF cycles was due to its adverse effect on the endometrium instead of oocytes damage.^27^ As all patients underwent FET after COH, we can regardless of the adverse impact of the high progesterone level on the endometrium and the corpus luteum function.

Utrogestan was usually used to support the luteal function in previous studies,^28^ thus, no direct evidence demonstrated the role of Utrogestan on oocyte development potential in previous reports. In our clinic, more than 500 children were born from luteal-phase ovarian stimulation, demonstrating that a natural high progesterone status in COH did not increase the risk of congenital malformations. In this trial, 13 healthy babies were born, which verified that elevated progesterone levels originating from Utrogestan had no detrimental effect on embryos and pregnancy. However, more researches were needed to confirm the long-term safety for children conceived using Utrogestan during COH.

Currently, it is debatable whether hCG or GnRH-a is the optimal preparation to use for triggering in traditional downregulation regimen.^29^ At the initiation stage of this trial, only GnRH-a 0.1 mg was used to trigger final oocyte maturation in the study group. Two cases out of 92 presenting LH levels lower than 15 IU/L (23) at the time of approximately 10 hours after trigger after by GnRH-a (13.01 and 13.87 IU/L) with a rate of mature oocytes 100% and 87.5%, respectively. Then, hCG was used to trigger oocyte maturation in case of poor results with low LH described in previous studies.^30,31^ GnRH-a was still used at present for the advantage of triggering comparable to the surge of the natural cycle and the elimination of OHSS.^32^ Once LH was below 1 IU/L, hCG was recommended to be employed based on our experiences (data unpublished). The viable embryo rate per oocyte retrieved after trigger by hCG was significant higher than that trigger by GnRH-a, which perhaps was correlated with the significances in hormone profiles, and other cycle characteristics of the 2 subgroups were comparable in our study. These were the preliminary data and rigorous researches, and further researches were needed to determine the way for ovulation in such a novel regimen.

Compared with GnRH agonist and antagonists, Utrogestan has the advantages of oral administration, user convenience, and fee reduction. Nevertheless, a major limitation of our study is the retrospective design of this study. In addition, the usage of Utrogestan and trigger drugs (hCG and GnRH-a) was still inexperienced, and all initial data were included in the study, which may contribute to the bias.

This retrospective trial confirmed for the first time that the oral delivery of Utrogestan is an effective way to block premature LH surges in normal ovulatory women undergoing IVF/ICSI treatments, which may help establish a novel regimen of COH in combination with embryo cryopreservation. Further studies should be performed on large samples to confirm the feasibility of this regimen and optimize this novel protocol in many perspectives such as the optimal dose of Utrogestan, the time for delivery, the way for trigger, and the suitable population.
